# Pathological complete response after chemotherapy in initially unresectable distal cholangiocarcinoma

**DOI:** 10.1007/s12328-024-02084-w

**Published:** 2024-12-28

**Authors:** Toshihiro Nakayama, Hiroshi Nakano, Reika Matsushita, Tomoaki Hayakawa, Shimpei Takagi, Yuya Tanaka, Takahiro Ozaki, Tsunehisa Matsushita, Yasuhiro Sumi, Masayuki Takagi

**Affiliations:** 1 Department of Surgery, National Hospital Organization Shizuoka Medical Center, 762-1 Nagasawa, Shimizu, Sunto District, Shizuoka, 411-0904 Japan; 2 Department of Diagnostic Pathology, National Hospital Organization Shizuoka Medical Center, 762-1 Nagasawa, Shimizu, Sunto District, Shizuoka, 411-0904 Japan

**Keywords:** Distal cholangiocarcinoma, Conversion surgery, Para-aortic lymph nodes

## Abstract

Surgical resection is the only curative treatment for cholangiocarcinoma, but it is often diagnosed at advanced stages, making surgical resection infeasible. Recently, the concept of conversion surgery has expanded the indications for surgical treatment, thanks to advancements in both perioperative management and chemotherapy. However, it remains unclear which patients benefit most from this treatment strategy. We present a case of initially unresectable cholangiocarcinoma in which a pathologic complete response was achieved following chemotherapy. A man in his seventies presented with jaundice and was referred to our hospital. Abdominal computed tomography revealed dilation of the intrahepatic bile ducts and thickening of the common bile duct, suggestive of distal cholangiocarcinoma. The tumor was initially unresectable due to metastatic para-aortic lymph nodes, and chemotherapy with gemcitabine and cisplatin was initiated. After six courses of chemotherapy, the lymph nodes showed a partial response, and tumor markers returned to normal levels. However, further chemotherapy was intolerable due to thrombocytopenia. Our cancer board then decided to perform a pancreaticoduodenectomy. Pathologic examination of the resected specimen showed complete disappearance of the primary tumor, but viable cancer cells were found in the resected lymph nodes. Seven months post-surgery, recurrence in the para-aortic nodes was detected through imaging and elevated tumor markers. Despite this, the patient remains alive 16 months post-surgery with normal tumor marker levels, following additional chemotherapy. Pathologic complete response of the primary tumor is rarely observed in patients with initially unresectable distal cholangiocarcinoma, and a multidisciplinary approach, including conversion surgery, may be effective in such cases.

## Introduction

Cholangiocarcinoma, which includes intrahepatic cholangiocarcinoma (ICC), distal cholangiocarcinoma (DCC), perihilar cholangiocarcinoma, and gallbladder cancer, is a rare type of cancer associated with a notably poor prognosis due to the difficulty of early detection [1]. Surgical resection is the only potential curative treatment [2], and the effectiveness of chemotherapy for unresectable disease with distant metastases remains limited [3].

Recent advancements in chemotherapy, surgical techniques, and perioperative management have significantly reduced perioperative mortality and morbidity for cholangiocarcinoma [2] and other cancers. This has led to the development of a therapeutic strategy known as “conversion surgery”, which aims to make previously unresectable tumors resectable using systemic treatments to downstage or downsize tumors [4]. Conversion surgery was initially adopted for managing gastric and pancreatic neoplasms [5], and its application in cholangiocarcinoma cases remains comparatively rare [6]. We hereby report a case of initially unresectable DCC in which conversion surgery became feasible after chemotherapy, leading to a pathologic complete response of the primary tumor.

## Case report

**Fig.1:**
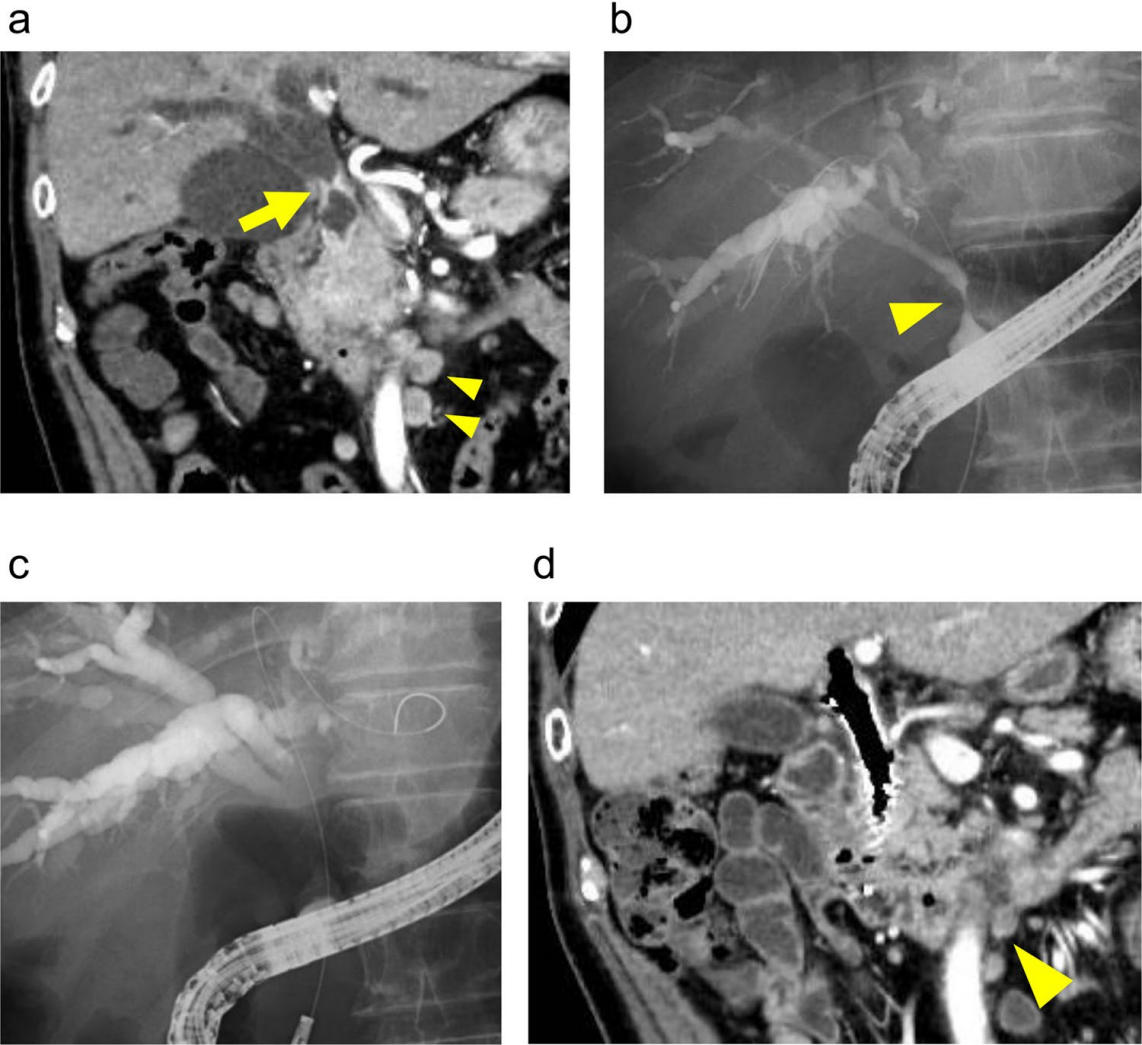
Preoperative abdominal MDCT and images of ERCP. **a** An abdominal MDCT showed a thickening common bile duct (arrow) and swollen para-aortic lymph nodes (arrowheads). **b** Narrowing of distal bile duct was seen in the first ERCP (arrowhead). **c** A guidewire was inserted in the left hepatic duct and the common bile duct during the first ERCP. **d** An abdominal MDCT after six courses of chemotherapy showed shrinkage of para-aortic lymph nodes (arrowhead). *MDCT* multidetector computed tomography, *ERCP* endoscopic retrograde cholangiopancreatography

**Fig. 2:**
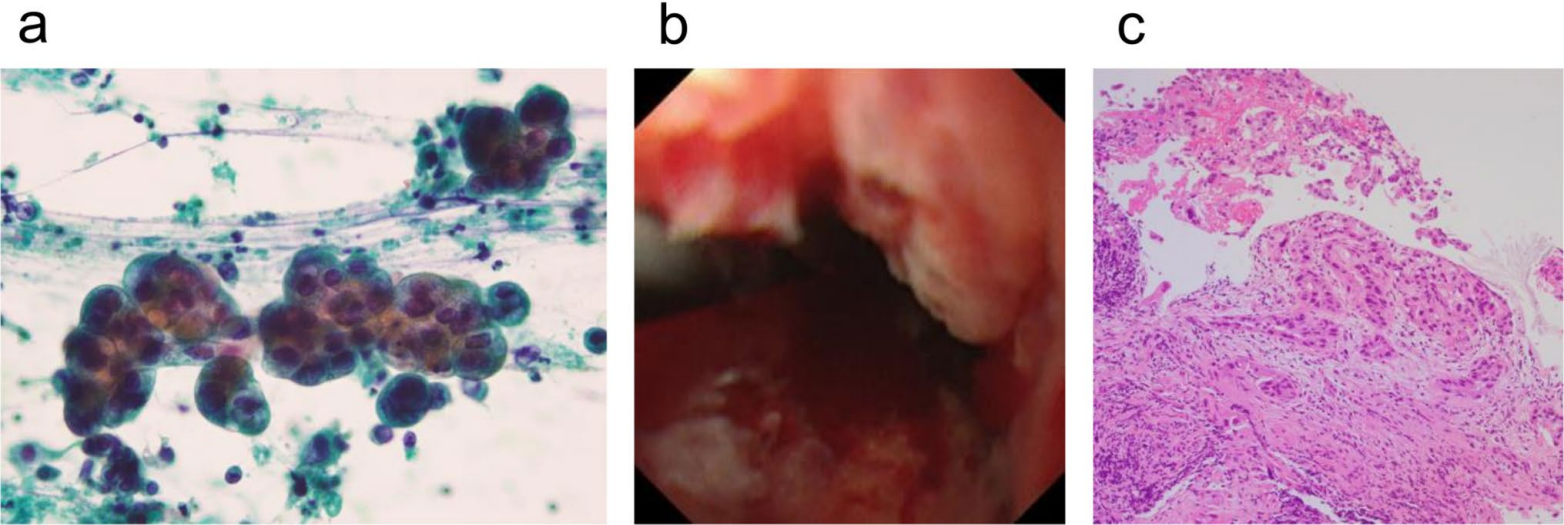
Pathologic findings leading to the diagnosis of cholangiocarcinoma before surgery. **a** Cytology showed class V cells. Atypical cells displaying nuclear pleomorphism were observed in a papillary cluster. The nuclei were eccentrically located, and the nuclear contours were irregular. **b** Peroral cholangioscopy showed significant narrowing of the bile duct consistent with malignancy. A mass with an irregular surface was noted within the narrowed segment. **c** Bile duct biopsy via cholangioscopy showed proliferation of atypical epithelial cells

A man in his seventies was referred to our hospital with a 2 week history of jaundice. His past medical history included appendicitis. Upon admission, laboratory tests revealed elevated levels of total bilirubin [10.6 mg/dl (reference range: 0.3–1.2 mg/dl)], direct bilirubin [7.1 mg/dl (reference range: 0.0–0.3 mg/dl)], and carbohydrate 19–9 (CA 19–9) [808.7 U/ml (reference range: 0.0–37.0 U/ml)]. Abdominal computed tomography (CT) showed narrowing of the common bile duct and swelling of the hilar and para-aortic lymph nodes, raising concerns for DCC. (Fig. 1a). Positron emission tomography-CT was not available at our hospital, and endoscopic ultrasound-guided fine needle aspiration was considered difficult for the para-aortic lymph nodes. Endoscopic retrograde cholangiopancreatography (ERCP) performed for cytology and preoperative biliary drainage revealed a narrowed distal bile duct (Fig. 1b, c). Biliary endoscopic sphincterotomy was performed. Cytologic analysis showed the presence of class V cells (Fig. 2a). Pathologic analysis of a bile duct biopsy obtained via peroral cholangioscopy confirmed a diagnosis of bile duct adenocarcinoma (Fig. 2b, c). The clinical stage was cT3N1M1, cStageIV, according to the TNM classification of malignant tumors (eighth edition) edited by the Union for International Cancer Control. As the patient was initially diagnosed as unresectable DCC, a metallic stent was placed in the common bile duct, and chemotherapy with gemcitabine (GEM) (1000 mg/m^2^) and cisplatin (CDDP) (25 mg/m^2^) was started.

**Fig. 3:**
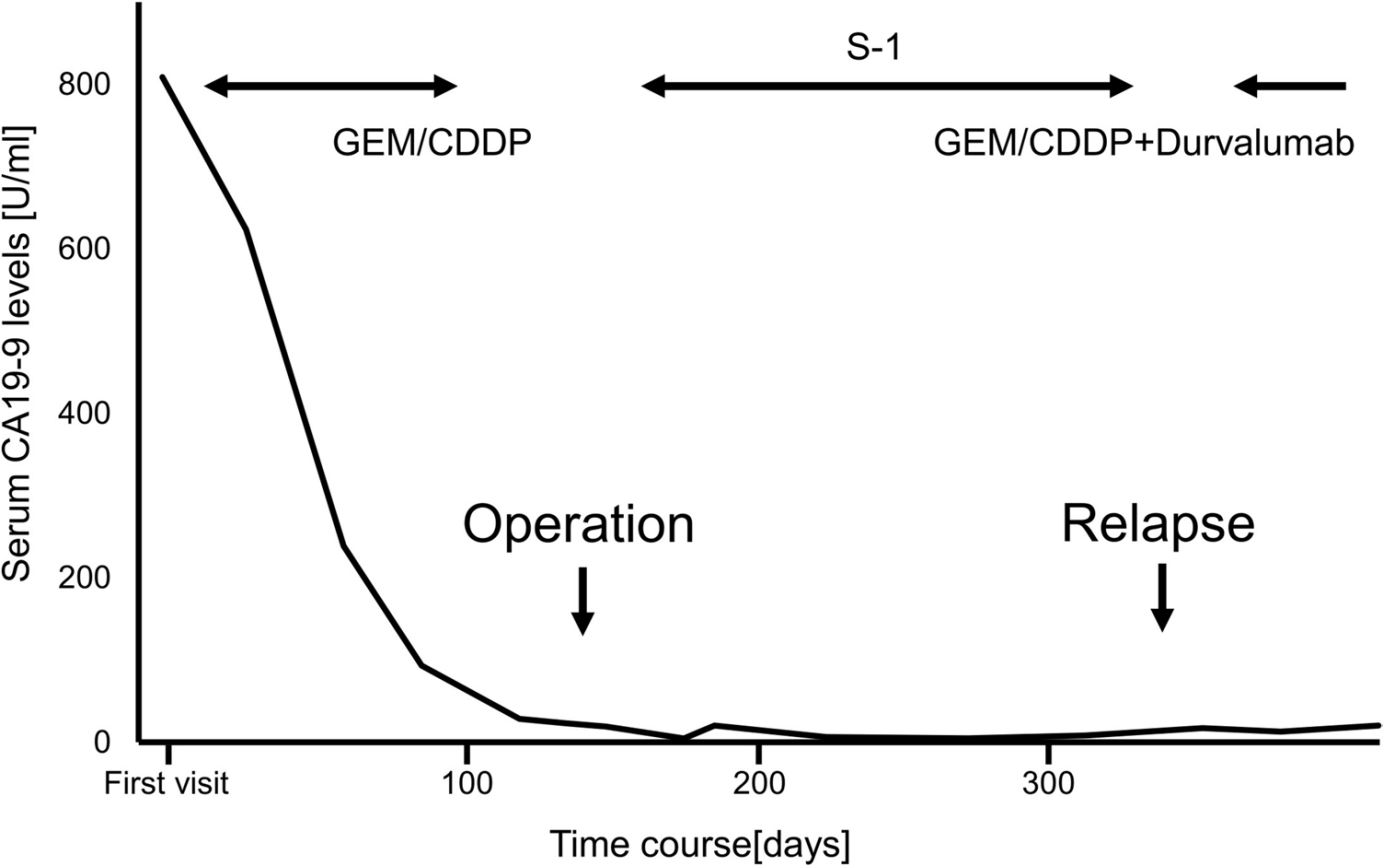
Serum CA 19–9 levels over the course of the treatment. The CA 19–9 levels dramatically decreased by initial chemotherapy using GEM and CDDP and remained within the normal range after the surgery. Systemic chemotherapy using GEM, CDDP and durvalumab was initiated after relapse. *CA 19–9* carbohydrate 19–9, *CDDP* cisplatin, *GEM* gemcitabine

After six courses of chemotherapy, our cancer board decided to proceed with conversion surgery for the following three reasons: reduction in the size of lymph nodes on abdominal CT, normalization of CA 19–9 levels, and the development of thrombocytopenia due to chemotherapy. According to the 7th edition of the General Rules for Clinical and Pathological Studies on Cancers of the Biliary Tract by the Japanese Society of Hepato-Biliary-Pancreatic Surgery, the hilar node at station 12 decreased in size from 25 to 13 mm, and the para-aortic node at station 16 decreased from 14 to 8 mm (Fig. 1d). The tumor demonstrated a partial response based on the Response Evaluation Criteria in Solid Tumors version 1.1. CA 19–9 levels decreased significantly from 808.7 U/ml initially to 22.9 U/ml after chemotherapy (Fig. 3). The platelet count after six courses was 66,000/mm^3^, which corresponds to grade 2 thrombocytopenia according to the Common Terminology Criteria for Adverse Events version 5.0, making further chemotherapy infeasible. It is important to note that the patient did not meet our standard criteria for conversion surgery in cholangiocarcinoma, specifically, maintaining a CA19-9 level within the normal range for six months. However, due to the development of thrombocytopenia, which prevented further chemotherapy, the decision was made to proceed with conversion surgery rather than risk tumor progression due to prolonged discontinuation of chemotherapy.

**Fig. 4:**
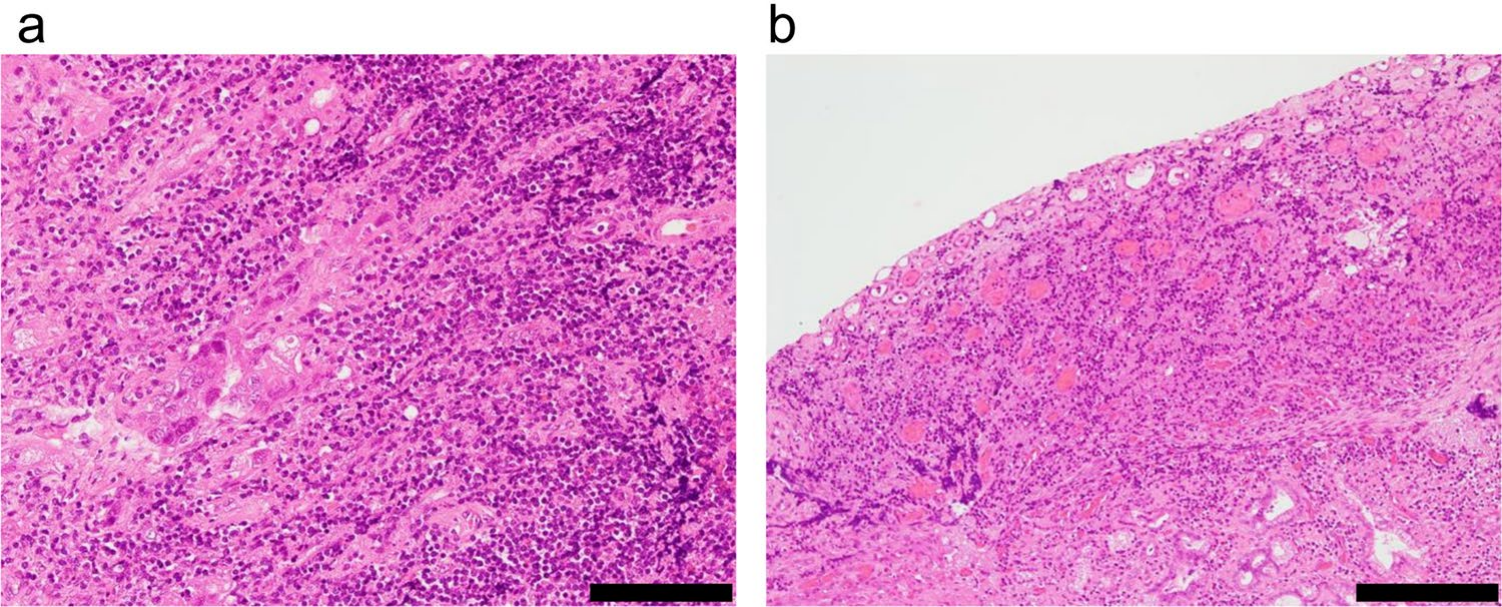
Pathological findings of the specimen. **a** Degenerated adenocarcinoma cells are detected in the lymph nodes. Scale bar = 100 μm. **b** Granulation tissue has formed in the lumen of the bile duct and cancer cells have disappeared. Scale bar = 100 μm

The patient underwent pancreaticoduodenectomy with D2 lymph node dissection 5 months after initially presenting to our hospital. Upon laparotomy, no ascites or peritoneal dissemination was observed, but para-aortic lymph node swelling was evident macroscopically, and para-aortic lymph node dissection was performed. A pancreaticogastrostomy was performed using the double purse-string telescoped method [7]. The operative time was 7 h and 10 min, with an estimated blood loss of 937 ml. Pathologic analysis revealed the complete disappearance of cancer cells at the primary site, indicating a complete response of the primary tumor due to preoperative chemotherapy (Fig. 4). Lymph node metastasis was confirmed at station 12 (2/4) and station 16 (2/8). The final diagnosis was distal bile duct adenocarcinoma classified as ypT0N1M1, ypStage IV. The postoperative course was uneventful, and the patient was discharged on postoperative day 15. Adjuvant chemotherapy with S-1 (100 mg/day) was administered for 6 months. Follow-up magnetic resonance imaging seven months after the surgery detected para-aortic lymph node metastasis. Our cancer board committee then decided to initiate systemic chemotherapy with GEM (1000 mg/m^2^), CDDP (25 mg/m^2^), and durvalumab (1500 mg/body) (GCD), which began eight months after surgery. Although durvalumab was not covered by national insurance preoperatively for this patient, it became available upon recurrence. Since thrombocytopenia forced the patient to discontinue chemotherapy before surgery, the treatment regimen was reduced to 80% of the dose, and further reduced to 65% of the dose due to grade 3 neutropenia. After nine courses of GCD therapy following recurrence, the tumor showed a partial response, and the regimen was switched to Durvalumab monotherapy. The patient is currently alive 16 months after the surgery without evidence of progression on imaging, and tumor markers remain within the normal range.

## Discussion

We presented a case of initially unresectable DCC due to distant lymph node metastasis, in which a pathologic complete response of the primary tumor was achieved after preoperative chemotherapy.

Recently, with advancements in chemotherapy, conversion surgery has emerged as a potential option for patients with clinically positive para-aortic nodes that respond to treatment. Two recent studies on cases with distant metastases have shown promising outcomes, particularly in patients who did not have pathologic distant metastases. In 2020, Noji et al. published a multi-center study involving 24 patients with initially unresectable biliary malignancies who underwent conversion surgery after at least 3 months of chemotherapy that resulted in stable disease or partial response [8]. The overall 5-year survival rate following surgery was 38.2%, with a median survival time of 34.3 months. This was significantly better than the 1.3% survival rate in the group that received chemotherapy alone, while the perioperative mortality was 4.2%. Similarly, a Korean study by Oh et al. reported on 12 patients who underwent conversion surgery for initially unresectable extrahepatic biliary tract cancer [9]. R0 resection was achieved in all cases, and the overall 5-year survival rate was 55.0%. The indications and optimal timing for conversion surgery have not yet been fully established. Based on the articles above, sustained clinical response to chemotherapy, such as reduction in tumor size or CA 19–9, might be considered as broad criteria for conversion as we await the further large-scale studies to confirm the validity of these criteria. The present case met the criteria by Noji et al., and was treated successfully with conversion surgery [8].

Pathologic complete response of the primary tumor is a rare finding in conversion surgery for cholangiocarcinoma, with only 14 reported cases, including our own, to date (Table 1) [9–21]. Of these, 9 cases involved ICC. This may be because the clinical response of the primary tumor is more easily evaluated in ICC compared to perihilar or distal cholangiocarcinoma, where stents are often placed in the common bile duct before chemotherapy, making it difficult to assess changes in the bile duct thickness on CT scans. For cases involving DCC, the indications for conversion surgery included a reduction in the size of lymph node metastasis by more than 50% or the disappearance of liver metastasis. The majority of cases were treated with chemotherapy regimens that included GEM. In 10 cases, more than 5 courses of preoperative chemotherapy were administered. Most cases achieved survival beyond 6 months. The effects of pathologic complete response on survival have not been fully elucidated in cholangiocarcinoma. Furthermore, with the recent introduction of new chemotherapy regimens, such as GEM, CDDP combined with durvalumab [22], or GEM, CDDP combined with S-1 [23], further research in this area is highly anticipated.

**Table 1:** Summary of previous cholangiocarcinoma cases with pathological complete response

Reference	Year	Age	Sex	Tumor type	Preoperative chemotherapy	Procedures	Survival
Watanabe	2017	70	F	DCC	GEM + S-1 (32 courses)	PPPD	> 48 months
Adachi	2019	69	F	DCC	GEM + CDDP (10 courses)	PPPD	> 19 months
Present case	2024	73	M	DCC	GEM + CDDP (6 courses)	PPPD	> 16 months
Oh	2021	57	F	PHCC	GEM + CDDP (40 courses)	Right hepatectomy caudate lobectomy	> 73 months
Slupski	2007	67	M	ICC	GEM + oxaliplatin (4 courses), GEM + CDDP (5 courses)	Left trisegmentectomy caudate lobectomy	> 6 months
Kato	2015	33	M	ICC	Doxorubicin, CDDP, 5-FU, IFN K (9 courses)	Right hepatectomy	> 30 months
Tran	2015	60	M	ICC	GEM + CDDP (4 courses)	Left trisegmentectomy caudate lobectomy	> 7 years
Matsubara	2016	68	F	ICC	GEM + CDDP + S-1 (12 courses)	Right hepatectomy caudate lobectomy	> 9 months
Tatsuguchi	2018	72	M	ICC	GEM + S-1 (10 courses)	Left hepatectomy	> 6 months
Abudalou	2021	47	M	ICC	GEM + CDDP + nabPTX (3 courses), Pembrolizumab (3 months)	Left hepatectomy	> 6 months
Sumiyoshi	2022	66	F	ICC	GEM + S-1 (4 courses)	Left trisegmentectomy caudate lobectomy	> 6 months
Zhang	2022	36	M	ICC	GEM + CDDP + nabPTX + Camrelizumab (5 courses)	n/a	> 1 month
Shibata	2023	76	M	ICC	GEM + CDDP (20 courses)	Extended posterior segmentectomy and partial liver resection	> 15 months
Shimamaki	2024	79	F	ICC	GEM + CDDP (4 courses)	Left hepatectomy caudate lobectomy	> 6 months

*CDDP* cisplatin, *DCC* distal cholangiocarcinoma, *GEM* gemcitabine, *ICC* intrahepatic cholangiocarcinoma, *IFN-K* interferon alpha Kinoid, *n/a* not available, *nabPTX* nab-paclitaxel, *PHCC* perihilar cholangiocarcinoma, *PPPD* pylorus-preserving pancreaticoduodenectomy, *5-FU* 5-fluorouracil

In conclusion, a pathologic complete response of the primary tumor was achieved in a patient with initially unresectable DCC. Despite the presence of lymph node metastases after chemotherapy, the conversion surgery appeared to be effective in this patient.

## Abbreviations

CA 19-9: Carbohydrate antigen 19–9
CDDP: Cisplatin
CT: Computed tomography
DCC: Distal cholangiocarcinoma
ERCP: Endoscopic retrograde cholangiopancreatography
GEM: Gemcitabine
ICC: Intrahepatic cholangiocarcinoma
PAN: Para-aortic lymph node
